# Polyphenol-Rich *Oenanthe javanica* as a Cardioprotective Functional Food Candidate Exhibiting Antiplatelet Activity via Suppression of Ca^2+^ Mobilization and Thromboxane A_2_ Production

**DOI:** 10.3390/ijms27125326

**Published:** 2026-06-12

**Authors:** Min-Kyu Park, Hyeonha Jang, Sung-Hun Choi, Jeong-Soo Bae, Jae-Ho Shin, Hwa-Jin Park

**Affiliations:** 1 Department of Applied Biosciences, Kyungpook National University, Daegu 41566, Republic of Korea; pmk601313@knu.ac.kr; 2 KNU NGS Core Facility, Kyungpook National University, Daegu 41566, Republic of Korea; 3 Microbalance Inc., Daegu 41566, Republic of Korea; 4 College of Pharmacology, Kyungpook National University, Daegu 41566, Republic of Korea; 5 Department of Biomedical Laboratory Science, College of Healthcare Medical Science and Engineering, Inje University, 197 Inje-ro, Gimhae 50834, Republic of Korea; 6 Cardiovascular Laboratory, Medical Center of Dong-A University, 26 Daesingongwon-ro, Busan 49201, Republic of Korea

**Keywords:** *Oenanthe javanica* (Blume) DC, polyphenols, platelet aggregation, intracellular Ca^2+^ mobilization, thromboxane A_2_, caffeic acid, chlorogenic acid, functional food, cardiovascular disease

## Abstract

Polyphenol-rich *Oenanthe javanica* (Blume) DC. is widely consumed in Asia but its impact on platelet activation, a cause of cardiovascular disease (CVD), is unclear. Collagen-driven platelet activation requires intracellular Ca^2+^ mobilization and thromboxane A_2_ (TXA_2_) production—rational targets for CVD prevention. A hot water extract of *O. javanica* (OJWE) was profiled by HPLC and tested on collagen-stimulated washed human platelets. Aggregation, Fura-2 [Ca^2+^]_i_, and TXB_2_ were measured, including combinations with verapamil, aspirin, caffeic acid (CA), and chlorogenic acid (CGA). Coagulation (PT/APTT) and ex vivo effects in Sprague–Dawley rats given OJWE (1 or 5 mg day^−1^, 30 days) were also evaluated. OJWE dose-dependently inhibited collagen-induced aggregation in a Ca^2+^-dependent manner, synergizing with verapamil, and suppressed [Ca^2+^]_i_ mobilization and TXA_2_ production. CA and CGA synergistically enhanced aspirin-mediated COX-1 inhibition. PT/APTT were unaffected in vitro and ex vivo. Dietary OJWE modestly but significantly reduced rat platelet aggregation without altering coagulation. OJWE attenuates platelet activation by selectively targeting Ca^2+^ mobilization and TXA_2_ biosynthesis without compromising hemostasis, supporting *O. javanica* as a functional food with cardiovascular potential at dietary intake.

## 1. Introduction

Platelet activation, including aggregation, granule secretion, and shape change, occurs when collagen and other subendothelial extracellular matrix components (i.e., von Willebrand factor and fibronectin) are exposed at sites of vascular injury. This process is regulated by intracellular Ca^2+^ mobilization and thromboxane A_2_ (TXA_2_) production, contributing to cardiovascular disorders such as thrombosis, atherosclerosis, and myocardial infarction [1,2,3].

Mobilized Ca^2+^ is essential for activating Ca^2+^-dependent phospholipase A_2_ (PLA_2_) and phospholipase C_γ2_ (PLC_γ2_), leading to the production of arachidonic acid (AA), the substrate for TXA_2_. It also activates the Ca^2+^-dependent myosin light chain kinase (MLCK), promoting platelet activation, including aggregation, granule secretion, and shape modification [4,5,6,7,8].

TXA_2_ further amplifies platelet activation by binding to its receptor on resting platelets and inducing vasoconstriction in both autocrine and paracrine manners, facilitating thrombus formation [9,10,11,12]. Consequently, agents that suppress collagen-induced Ca^2+^ mobilization and subsequent TXA_2_ production are recognized as promising antithrombotic modulators.

*Oenanthe javanica* (Blume) DC., commonly known as water dropwort (or *Mi-na-ri* in Republic of Korea), thrives in wetlands and waterways. This plant is widely consumed as a vegetable in East and Southeast Asia and has been utilized in traditional Chinese medicine for centuries [13]. Herbal tea and ethanol extracts prepared from, along with its constituents—phenolic acids and flavonoids—reportedly exhibit antioxidant [14], anti-inflammatory [15,16], hepatoprotective [16], and anti-coagulant activities [17,18]. However, research indicates that *O. javanica* extracts do not prolong prothrombin time (PT) or activated partial thromboplastin time (APTT) ex vivo [19], leaving its coagulation-related effects ambiguous.

Currently, no study has systematically assessed the impact of *O. javanica* on collagen-induced platelet activation markers, such as intracellular Ca^2+^ and TXA_2_. Collagen acts as a thrombogenic substrate at vascular injury sites, initiating robust platelet activation primarily through glycoprotein VI-dependent Ca^2+^ mobilization and TXA_2_ generation, rendering it a physiologically relevant model for investigating Ca^2+^–TXA_2_-driven platelet activation [20,21,22,23].

**Figure 1:**
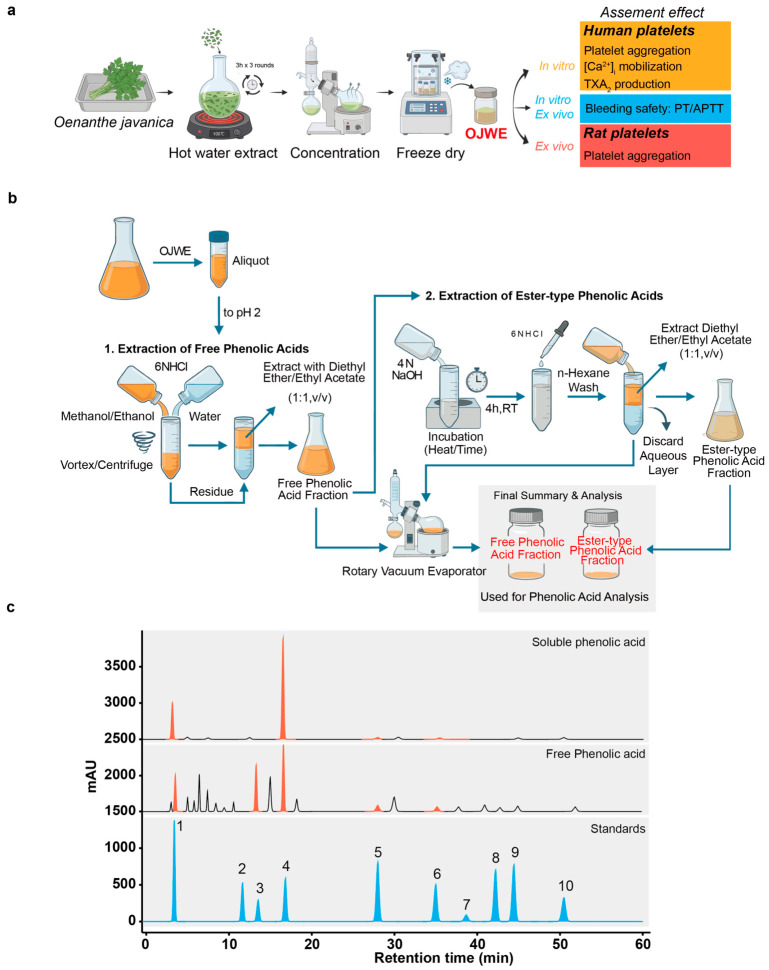
Experimental design, fraction preparation, and HPLC chromatogram for analyzing phenolic compounds in OJWE. (**a**) Experimental design to assess the effects of *O. javanica* on CVD risk. (**b**) Procedure for extracting and separating free and esterified phenolic acids from OJWE. (**c**) HPLC chromatogram of phenolic compounds in OJWE: (1) gallic acid; (2) 4-hydroxybenzoic acid; (3) caffeic acid; (4) chlorogenic acid; (5) p-coumaric acid; (6) ferulic acid; (7) naringenin; (8) quercetin-3-galactoside; (9) salicylic acid; and (10) o-coumaric acid. HPLC, high-performance liquid chromatography; CVD, cardiovascular disease; OJWE, hot water extracts from *O. javanica*; DE, diethyl ether; EA, ethyl acetate; GA, gallic acid; CA, caffeic acid; CGA, chlorogenic acid; RT, room temperature.

Therefore, this study aimed to determine whether the hot water extract of *O. javanica* (OJWE) downregulates thrombogenic molecules such as intracellular Ca^2+^ and TXA_2_ in collagen-activated human platelets in vitro, while also examining its effect on ex vivo platelet aggregation without altering PT or APTT (Figure 1a).

## 2. Results

### 2.1. Total Phenol and Phenolic Compound Content in OJWE

The total phenol content in *O. javanica* was measured at 30.1 ± 1.5 mg GAE per gram of OJWE, resulting in a yield of 2.3% (Table S1).

Phenolic compounds in OJWE were identified using high-performance liquid chromatography (HPLC, Figure 1c). OJWE was abundant in phenolic compounds, including gallic (GA), caffeic (CA), and chlorogenic (CGA) acids. As detailed in Table S2, these standards were detected at retention times of 3.99 ± 0.14, 14.74 ± 0.60, and 17.75 ± 0.09 min, respectively. In the soluble phenolic acid ester fraction from OJWE, GA and CGA appeared at 4.14 ± 0.14 and 17.57 ± 0.14 min, respectively (Table S2). The free phenolic fraction exhibited GA, CA, and CGA at 4.14 ± 0.14, 14.14 ± 0.14, and 17.57 ± 0.14 min, respectively (Table S2). The retention times for GA, CA, and CGA in OJWE matched those of the standards, confirming their presence in the extract.

The contents of GA, CA, and CGA in OJWE were measured at 36.65, 47.75, and 200.80 μg mg-OJWE^−1^, respectively (Table S3). The GA (36.65 μg mg-OJWE^−1^) and CGA (200.80 μg mg-OJWE^−1^) values represent the combined amounts from the soluble phenolic acid ester fraction and free phenolics, respectively. Previous phytochemical studies of *O. javanica* have primarily employed organic solvent extracts, including 70% ethanol extracts, which generally maximize phenolic recovery [24]. In contrast, the present study utilized a hot-water extract to better reflect the traditional dietary consumption of *O. javanica*. Therefore, the phenolic profile of OJWE may provide information that is more relevant to dietary exposure than that obtained from solvent extracts. Notably, while *O. javanica* is known for its high flavonoid content [17], this study did not quantify flavonoids in OJWE.

### 2.2. Combinatorial Inhibitory Effects of OJWE on Collagen-Induced Human Platelet Aggregation

**Figure 2:**
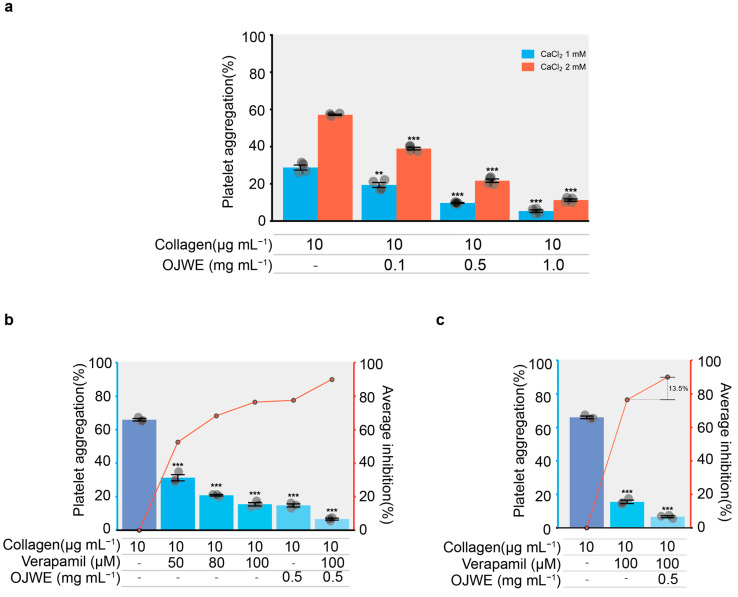
In vitro effects of OJWE and verapamil on collagen-induced human platelet aggregation. Human platelet aggregation was assessed as detailed in the Section 4, with monitoring via light transmission. (**a**) In vitro inhibitory effects of OJWE on collagen-induced platelet aggregation under extracellular Ca^2+^ conditions (1 and 2 mM). (**b**) In vitro combinatorial inhibitory effects of verapamil and OJWE on collagen-induced platelet aggregation. (**c**) Additive inhibitory effects of OJWE in conjunction with verapamil on collagen-induced platelet aggregation. OJWE, hot water extracts from *O. javanica*. Data are presented as the mean ± standard deviation (SD, *n* = 3). ** *p* < 0.01, *** *p* < 0.001.

Collagen (10 μg mL^−1^) induced platelet aggregation of 28.75% ± 2.65% and 57.08% ± 0.72% with 1 and 2 mM of extracellular CaCl_2_, respectively (Figure 2a). However, OJWE significantly inhibited collagen-induced extracellular Ca^2+^-dependent platelet aggregation in a dose-dependent manner (Figure 2a), suggesting that OJWE reduces platelet aggregation by impeding Ca^2+^ influx. Therefore, the combinatorial inhibitory effects of OJWE and verapamil, a Ca^2+^ influx blocker, on collagen-induced platelet aggregation were investigated.

### 2.3. Additive Inhibitory Effects of OJWE and Verapamil on Collagen-Induced Human Platelet Aggregation

Verapamil (50–100 μM), a Ca^2+^ influx blocker, inhibited collagen-induced human platelet aggregation dose-dependently in the presence of 2 mM CaCl_2_ (Figure 2b). When combined, verapamil (100 μM) and OJWE (0.5 mg mL^−1^) inhibited this aggregation by up to 90%, compared to the 76.5% inhibition by verapamil alone and 77.6% by OJWE alone (Figure 2b).

Specifically, OJWE (0.5 mg mL^−1^) enhanced the effect of verapamil (100 μM), resulting in a 90.0% inhibition of collagen-induced aggregation, while verapamil alone achieved 76.5% inhibition (Figure 2c). This indicates an additive inhibitory effect, with OJWE providing an additional 13.5% inhibition beyond that of verapamil (100 μM) alone (Figure 2c). Collectively, these findings suggest that OJWE mitigates collagen-induced platelet aggregation, likely by suppressing Ca^2+^ influx.

### 2.4. Inhibitory Effects of OJWE on Collagen-Induced Intensity of Fura-2 Binding to Intracellular Ca^2+^

**Figure 3:**
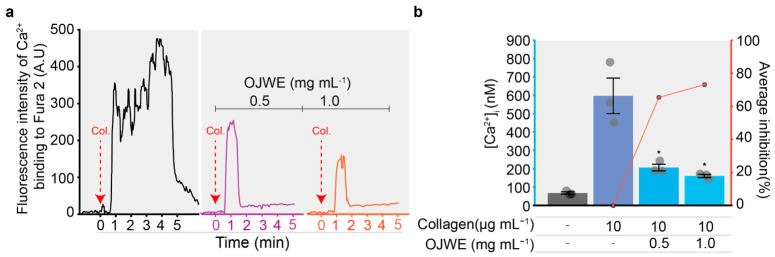
In vitro effects of OJWE on collagen-induced [Ca^2+^]_i_ mobilization. [Ca^2+^]_i_ mobilization was measured as described in the Section 4. (**a**) In vitro inhibitory effects of OJWE on Fura-2 fluorescence intensity associated with [Ca^2+^]_i_ mobilization by collagen. (**b**) In vitro inhibitory effects of OJWE on collagen-induced [Ca^2+^]_i_ mobilization. OJWE, hot water extracts from *O. javanica*. Col., Collagen. Data are presented as the mean ± SD (*n* = 3). * *p* < 0.05.

Fura-2 acetoxymethyl ester (Fura-2 AM), a cell-permeable and nonfluorescent Ca^2+^ probe, readily diffuses across the plasma membrane. Once inside the cytosol, endogenous esterases hydrolyze the AM groups, yielding the active, hydrophilic Fura-2 that is retained within the cell, thus allowing it to bind cytosolic free Ca^2+^ ([Ca^2+^]_i_) and increase fluorescence intensity. As shown in Figure 3a, collagen (10 μg mL^−1^) increased fluorescence intensity, implying that Fura-2 bound to the increased [Ca^2+^]_i_ induced by collagen, thereby increasing fluorescence intensity. Nevertheless, OJWE (0.5–1 mg mL^−1^) reduced collagen-induced fluorescence intensity in a dose-dependent manner (Figure 3a). This implies that OJWE reduced the intracellular calcium available for binding to Fura-2 by decreasing the collagen-induced [Ca^2+^]_i_ mobilization. In reality, OJWE reduced collagen-induced [Ca^2+^]_i_ mobilization (Figure 3b).

### 2.5. Inhibitory Effects of OJWE on Collagen-Induced [Ca^2+^]_i_ Mobilization

[Ca^2+^]_i_ mobilization was assessed by monitoring changes in the fluorescence of Ca^2+^-bound Fura-2 (Figure 3a). As illustrated in Figure 3b, collagen (10 μg mL^−1^) elevated [Ca^2+^]_i_ from a basal level of 67.6 ± 11.1 to 597.2 ± 167.5 nM in the presence of 2 mM CaCl_2_. Conversely, OJWE (0.5–2 mg mL^−1^) reduced collagen-induced [Ca^2+^]_i_ in a dose-dependent manner, achieving inhibition rates of 65.4%, 73.1%, and 80.4%, respectively.

### 2.6. Inhibitory Effects of OJWE on Collagen-Induced TXA_2_ Production

**Figure 4:**
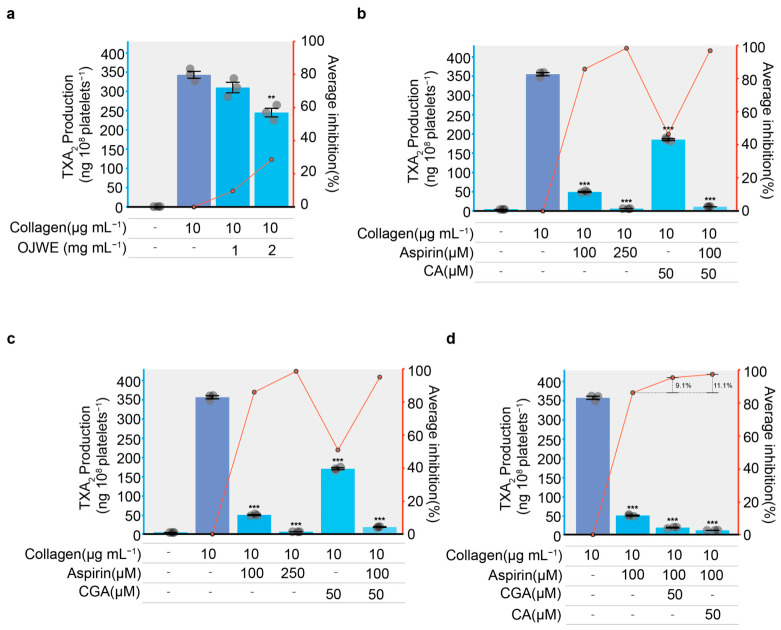
In vitro effects of OJWE on collagen-induced TXA_2_ production. TXA_2_ production was assessed as described in the Section 4. (**a**) In vitro inhibitory effects of OJWE on collagen-induced TXA_2_ production. (**b**) In vitro combinatorial inhibitory effects of aspirin and CA on collagen-induced TXA_2_ production. (**c**) In vitro combinatorial inhibitory effects of aspirin and CGA on collagen-induced TXA_2_ production. (**d**) In vitro additive inhibitory effects of CA and CGA, in conjunction with aspirin, on collagen-induced TXA_2_ production. In Figure 4d, additive inhibitory effects (%) of CGA to aspirin on TXA_2_ production = [Inhibitory effects (%) by CGA plus aspirin] − Inhibitory effects (%) by aspirin alone. Additive inhibitory effects (%) of CA to aspirin on TXA_2_ production = [Inhibitory effects (%) by CA plus aspirin] − Inhibitory effects (%) by aspirin alone. OJWE, hot water extracts from *O. javanica*; TXA_2_, thromboxane A_2_; CA, caffeic acid; CGA, chlorogenic acid. Data are presented as the mean ± SD (*n* = 3). ** *p* < 0.01, *** *p* < 0.001.

In resting platelets, TXA_2_ levels were 1.03 ± 0.06 ng per 10^8^ platelets. However, upon activation with collagen (10 μg mL^−1^), TXA_2_ production surged to 343.01 ± 15.63 ng per 10^8^ platelets (Figure 4a). OJWE significantly decreased TXA_2_ production in a dose-dependent manner, resulting in levels of 309.97 ± 23.67 and 244.97 ± 19.47 ng per 10^8^ platelets at concentrations of 1 and 2 mg mL^−1^, respectively (Figure 4a). This indicates that OJWE may inhibit cyclooxygenase-1 (COX-1) or TXA_2_ synthase, which are involved in TXA_2_ production in collagen-activated platelets. To further explore this, we examined the combined effects of OJWE with aspirin, a COX-1 inhibitor known to reduce TXA_2_ production.

### 2.7. Additive Inhibition of Collagen-Induced TXA_2_ Production by CA and CGA, OJWE Components, in Combination with Aspirin

Aspirin (100 and 250 μM), a COX-1 inhibitor, dose-dependently reduced collagen-elevated TXA_2_ levels (345.6 ± 7.5 ng per 10^8^ platelets) by 85.7% and 98.3%, respectively (Figure 4b). When CA (50 μM) was added with aspirin (100 μM) to the platelet aggregation system and activated with collagen (10 μg mL^−1^), TXA_2_ production was 11.2 ± 0.4 ng per 10^8^ platelets (Figure 4b), indicating an inhibition rate of 96.8% compared to collagen alone. This effect surpassed the inhibition rates of aspirin (85.7%) and CA (46.4%) individually (Figure 4b).

In a similar experiment involving CGA (50 μM) and aspirin (100 μM), TXA_2_ production was measured at 18.0 ± 1.9 ng per 10^8^ platelets (Figure 4c), corresponding to an inhibition rate of 94.8% against collagen-induced TXA_2_ levels (345.6 ± 7.5 ng per 10^8^ platelets, Figure 4c). This inhibition rate also exceeded that of aspirin (85.7%) and CGA (50.8%) alone (Figure 4c).

Consequently, CA and CGA from OJWE, in conjunction with aspirin, exhibited an additive effect in inhibiting collagen-induced TXA_2_ production, suggesting that they may similarly inhibit COX-1 activity.

CGA (50 μM) and CA (50 μM) from OJWE inhibited collagen-induced TXA_2_ production by up to 94.8% and 96.8%, respectively, in the presence of aspirin (100 μM), which alone inhibited TXA_2_ production by 85.7% (Figure 4d).

To further compare the magnitude of the additive effects, the additional inhibitory effects (%) of aspirin in combination with CGA or CA were calculated from the combinatorial inhibition data shown in Figure 4b,c. As summarized in Figure 4d, caffeic acid and chlorogenic acid additionally enhanced the inhibitory effect (85.7%) of aspirin on collagen-induced TXA_2_ production by 9.1% and 11.1%, respectively (Figure 4d, inset).

This suggests an additive effect, with CGA and CA providing additional inhibition (9.1% and 11.1%, respectively) beyond aspirin’s individual effect (Figure 4d). These findings indicate that CA and CGA reduce collagen-induced TXA_2_ production, likely by suppressing COX-1-dependent AA metabolism.

### 2.8. OJWE Does Not Prolong PT and APTT in Human Plasma In Vitro

**Figure 5:**
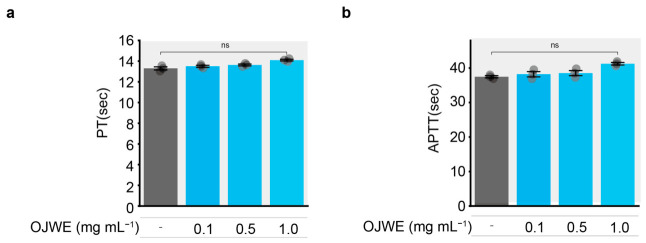
In vitro effects of OJWE on blood coagulation parameters. PT and APTT were assessed as described in the Section 4. (**a**) In vitro effects of OJWE on PT. (**b**) In vitro effects of OJWE on APTT. OJWE, hot water extracts from *O. javanica*; PT, prothrombin time; APTT, activated partial thromboplastin time. ns, not significant versus the control (*n* = 3).

A key concern with antiplatelet or antithrombotic agents is the risk of bleeding attributed to the disruption of normal hemostasis [25,26]. Considering that OJWE inhibits platelet aggregation (Figure 2a), we assessed its impact on the coagulation cascade, essential for physiological hemostasis. Therefore, PT and APTT, which reflect the extrinsic and intrinsic pathways, respectively, were measured. As illustrated in Figure 5a,b, OJWE did not significantly prolong PT or APTT (PT: 13.30 ± 0.26 s; APTT: 37.47 ± 0.55 s) compared to the controls. These results suggest that OJWE does not interfere with plasma coagulation pathways.

### 2.9. Effects of OJWE Administration on Daily Feed Intake, Body Weight Gain, and Feed Efficiency Ratio (FER)

As presented in Table 1, rats in the control group consumed 24.3 ± 1.5 g of feed daily, gaining 4.3 ± 0.4 g of body weight. In contrast, rats treated with OJWE (1 and 5 mg day^−1^) consumed 26.2 ± 2.1 and 26.8 ± 0.7 g daily, respectively, gaining 4.0 ± 0.4 and 4.4 ± 0.1 g. While feed intake was higher in the OJWE groups, body weight gain did not vary significantly. Furthermore, the FER was significantly lower in the OJWE groups (0.153 at 1 mg day^−1^ and 0.160 at 5 mg day^−1^) than in the control group (0.176).

### 2.10. Ex Vivo Inhibitory Effects of Dietary OJWE on Rat Platelet Aggregation

**Figure 6:**
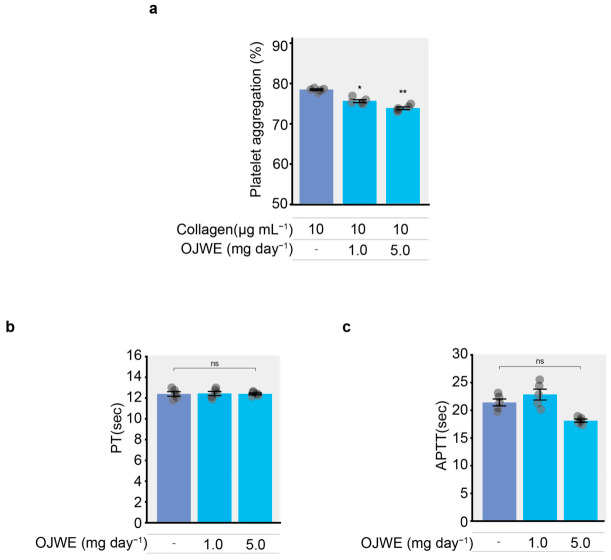
Ex vivo effects of dietary OJWE on rat platelet aggregation and blood coagulation parameters. Platelet aggregation was measured as described in the Section 4, with monitoring via light transmission. PT and APTT were determined as detailed in the Section 4. (**a**) Ex vivo inhibitory effects of dietary OJWE on collagen-induced rat platelet aggregation. (**b**) Ex vivo effects of dietary OJWE on PT. (**c**) Ex vivo effects of dietary OJWE on APTT. OJWE, hot water extracts from *O. javanica*; PT, prothrombin time; APTT, activated partial thromboplastin time. Data are expressed as the mean ± SD (n = 5). * *p* < 0.05, ** *p* < 0.01 versus the control. ns, not significant versus the control.

In rats fed a control diet for 30 days, collagen (10 μg mL^−1^) stimulation of platelets (10^8^ platelets mL^−1^) resulted in platelet aggregation increasing to 78.3% ± 1.1% (Figure 6a). In contrast, rats administered OJWE at 1 and 5 mg day^−1^ for 30 days exhibited a modest yet significant decrease in collagen-induced platelet aggregation to 75.5% ± 2.0% at 1 mg day^−1^ (*p* < 0.05) and 73.7% ± 1.8% at 5 mg day^−1^ (*p* < 0.001), respectively, demonstrating a dose-dependent effect (Figure 6a).

### 2.11. OJWE Does Not Prolong PT and APTT in Rat Plasma Ex Vivo

After administering OJWE to rats for 30 days, plasma was separated to assess its effects on blood coagulation. As depicted in Figure 6b, the PT for the control group was 12.40 ± 0.49 s, while the OJWE (1 mg day^−1^) group recorded 12.44 ± 0.45 s, and the OJWE (5 mg day^−1^) group yielded 12.40 ± 0.20 s, indicating no significant changes from the control. The APTT values for the control, OJWE (1 mg day^−1^), and OJWE (5 mg day^−1^) groups were 21.41 ± 1.40, 22.83 ± 2.19, and 18.13 ± 0.64 s, respectively, again yielding no significant differences from the control (Figure 6c). Overall, these results suggest that OJWE does not impair ex vivo plasma coagulation.

## 3. Discussion

Platelet activation within blood vessels is a key pathological event leading to thrombus formation [1,3]. Consequently, inhibiting platelet activation is vital for preventing and treating thrombosis [3,27]. This study demonstrates that phenolic-rich OJWE reduces collagen-induced platelet activation by suppressing [Ca^2+^]_i_ mobilization and TXA_2_ production. Collagen is a principal physiological agonist that facilitates platelet adhesion, activation, and procoagulant surface formation at vascular injury sites [28,29]; thus, inhibiting collagen-driven signaling is a mechanistically relevant strategy to prevent thrombus formation. OJWE significantly inhibited extracellular Ca^2+^ (1–2 mM)-dependent aggregation and reduced collagen-induced increases in cytosolic Ca^2+^ concentrations. Considering that the mobilization of intracellular Ca^2+^ is a central event linking glycoprotein VI engagement to downstream activation [4,5,6,7,8], this suppression likely underlies the inhibition of aggregation. Moreover, the synergistic enhancement with verapamil supports a Ca^2+^-channel-related mechanism.

The inhibition of [Ca^2+^]_i_ was accompanied by a significant decrease in TXA_2_ generation. TXA_2_, synthesized from AA via COX-1 and thromboxane synthase, acts as an autocrine/paracrine amplifier that boosts platelet recruitment and vasoconstriction [9,10,11,12]. OJWE inhibited TXA_2_ production in a dose-dependent manner, with its major phenolic constituents—CA and CGA—enhancing aspirin’s inhibition of TXA_2_, indicating a cooperative suppression of COX-1-dependent AA metabolism. Previous studies have demonstrated that both CA and CGA inhibit TXA_2_ production by reducing COX-1 activity, supporting this additive interaction [30,31]. Collectively, these findings suggest that OJWE targets two fundamental pathways sustaining platelet activation: Ca^2+^ mobilization and TXA_2_ biosynthesis. In this study, CGA and CA were selected as representative phenolic constituents of OJWE based on our previous findings demonstrating their inhibitory effects on platelet aggregation and TXA_2_ generation [30,31]. Future studies are warranted to determine whether other phenolic constituents, including GA, also contribute to the antiplatelet activity of OJWE.

The dietary administration of OJWE resulted in a dose-dependent reduction in ex vivo platelet aggregation, with statistically significant inhibition observed at both 1 (*p* < 0.05) and 5 (*p* < 0.001) mg day^−1^. Although the overall magnitude of inhibition (an approximately 3–5% reduction) was smaller than the inhibitory effects (62% at 0.5 mg mL^−1^) by OJWE observed in the in vitro experiments, these findings indicate that even low dietary doses of OJWE can attenuate platelet reactivity under physiological conditions.

Importantly, OJWE did not prolong PT or APTT in vitro or ex vivo, aligning with previous findings wherein *O. javanica* extracts exerted minimal effects on coagulation parameters [19]. Prolonged PT/APTT is linked to bleeding complications associated with diverse synthetic antiplatelet agents [25,26,32]. Therefore, the absence of coagulation impairment observed here indicates that OJWE minimally interferes with hemostatic capacity under the tested conditions.

The phenolic content of OJWE offers additional mechanistic insights. Based on administered doses of 1 and 5 mg per day, estimated intakes of CA were 1.425 and 7.125 mg over 30 days, while CGA intakes were 6.024 and 30.12 mg, respectively (Table S4). Rats receiving 1 or 5 mg of OJWE per day were estimated to absorb physiologically relevant quantities of CA (1.35–6.77 mg over 30 days) and CGA (1.99–9.94 mg over 30 days), based on reported intestinal absorption rates (approximately 95% and 33% for CA and CGA, respectively) [33]. Prior studies indicate that CA (1.25–5 mg kg^−1^) and CGA (0.1–1 mmol L^−1^) inhibit arteriolar thrombus formation in vivo [34,35], suggesting that the absorbed phenolics from dietary OJWE potentially contribute to the observed antiplatelet effects. Nonetheless, both acids undergo extensive metabolism in the gut microbiota and liver [36,37], producing bioactive metabolites that may also play a role in the observed activity.

It should also be noted that thermal extraction may influence the recovery and stability of phenolic compounds. Previous studies [38,39,40] have reported that heat treatment can induce degradation or transformation of thermosensitive phenolics, including hydroxycinnamic acid derivatives, while it is also reported that the total phenolics and total flavonoids in tomatoes were not changed by thermal processing [41]. Therefore, the phenolic composition of OJWE may differ from that of fresh *O. javanica*. Nevertheless, because OJWE was prepared using a hot-water extraction process that resembles traditional culinary preparation, the identified phenolic profile may provide information that is more relevant to actual dietary exposure and functional-food applications.

Most previous phytochemical studies of *O. javanica* used organic solvents, such as ethanol, chloroform, and ethyl acetate, to maximize the recovery of phenolic compounds [24,42]. While such approaches are useful for phytochemical profiling, they may not adequately reflect the forms in which *O. javanica* is commonly consumed in the diet. In contrast, the present study employed a hot-water extract because *O. javanica* is traditionally consumed as a cooked vegetable or in aqueous food preparations. Therefore, the phenolic composition identified in OJWE may provide phytochemical information that is more relevant to dietary exposure and functional-food applications. From this perspective, the predominance of CGA and CA observed in OJWE suggests that CGA and CA may represent a major bioaccessible phenolic constituents under dietary conditions. Accordingly, the antiplatelet activity of OJWE may have greater translational relevance than the results obtained solely from organic solvent extracts, because the extract preparation more closely reflects actual human consumption patterns. Furthermore, confirming how these substances affect the antiplatelet activity of OJWE through the analysis of OJWE’s GA and unidentified substances will be helpful in enhancing the antiplatelet action of *O. javanica*.

### Relevance to Dietary Consumption and Functional Food Applicability

*O. javanica* is a widely consumed vegetable in East Asian diets, featured in soups, blanched greens, fresh salads, and juices [17,19,43,44]. Typical culinary portions are estimated to be several tens of grams per serving, providing a realistic context for habitual phenolic intake [45]. When adjusted for body surface area, the OJWE doses used in rats (1–5 mg day^−1^; approximately 0.004–0.02 mg g^−1^ body weight) correspond to approximately 0.05–0.25 mg kg^−1^ for a 60 kg human [46], which is within the range achievable through normal culinary consumption. Considering that OJWE was prepared using simple hot water extraction—comparable to common cooking methods such as blanching or soup preparation—the experimental extract reflects physiologically plausible dietary exposure [47,48].

Moreover, the estimated absorbed amounts of CA and CGA fall within ranges associated with biological activity in vivo [49], suggesting that regular consumption of *O. javanica* could feasibly influence platelet reactivity in humans. Culinary factors such as boiling time, food matrix, and preparation method may influence the yield and bioavailability of phenolics, warranting further studies to determine how typical dietary patterns modify the functional efficacy of *O. javanica* [50,51,52].

## 4. Materials and Methods

### 4.1. Materials

*O. javanica* (Chung-Do, Kyung Buk, Republic of Korea) was purchased from a local market. Aspirin, verapamil hydrochloride (verapamil HCl), CA, and CGA were procured from Sigma Chemical Co., Ltd. (St. Louis, MO, USA). Collagen was sourced from Chrono-Log Corporation (Havertown, PA, USA), and other reagents were acquired from Sigma Chemical Co., Ltd. (St. Louis, MO, USA). Fura-2 AM was also purchased from Sigma Chemical Co., Ltd. (St. Louis, MO, USA). The thromboxane B_2_ (TXB_2_) enzyme immunoassay (EIA) kit was procured from GE Healthcare (Buckinghamshire, UK). PT and APTT assay reagents were sourced from Fisher Diagnostics (Middletown, VA, USA).

### 4.2. Preparation of O. javanica Hot Water Extract

*O. javanica* (1 kg) was thoroughly washed, finely chopped, and placed in distilled water. The extract underwent three rounds of hot water extraction at 100 °C for 3 h each. The hot water extract was subsequently centrifuged at 125× *g* for 30 min using a high-speed refrigerated centrifuge (Hanil Co., Ltd., Seoul, Republic of Korea). The supernatant was filtered through Whatman No. 4 filter paper (Maidstone, UK). The filtrate was concentrated with a rotary vacuum evaporator (Eyela, Type N-Nm, Tokyo Rikakikai Co., Ltd., Tokyo, Japan) at room temperature and subsequently freeze-dried (Il-Shin-BioBase, Dongducheon-si, Republic of Korea). The dried mass (OJWE) weighed 22.82 g, resulting in a 2.3% recovery (Table S1). The extract was stored at −20 °C until required for experimentation.

### 4.3. Measurement of Total Phenol Content in OJWE

Total phenol content was measured using a modified method from Singleton and Maria [53,54]. A volume of 200 μL (50, 100, or 200 μg) of OJWE dissolved in distilled water was combined with 1 mL of 10% Folin–Ciocalteu phenol reagent. The mixture was vortexed thoroughly and allowed to sit at room temperature for 3 min. Thereafter, 0.8 mL of 7.5% Na_2_CO_3_ solution was added, mixed thoroughly, and left at room temperature for 30 min. The mixture was centrifuged at 125× *g* for 10 min, and the supernatant was collected. Absorbance was measured at 765 nm, with a standard curve prepared using GA in the same manner as the sample solution.

### 4.4. Fractionation of Free Phenolic Acids and Soluble Phenolic Acid Esters

Free and ester-type phenolic acid fractions were separated from OJWE using Krygier’s method [55]. OJWE was acidified to pH 2 with 6 N HCl, washed three times with an equal volume of n-hexane, and subsequently extracted with diethyl ether/ethyl acetate (1:1, *v*/*v*) to obtain the free phenolic acid fraction. The remaining aqueous layer was hydrolyzed with 4 N NaOH at room temperature for 4 h, adjusted to pH 2 with 6 N HCl, washed with n-hexane, and extracted with diethyl ether/ethyl acetate (1:1, *v*/*v*) to yield the ester-type phenolic acid fraction. The diethyl ether/ethyl acetate fractions of free and esterified phenolic acids were concentrated using a rotary vacuum evaporator at room temperature and prepared for phenolic acid analysis. A representative diagram is presented in Figure 1b.

### 4.5. HPLC Detection and Analysis of Phenolic Compounds

The diethyl ether/ethyl acetate (1:1, *v*/*v*) fraction derived from OJWE, as illustrated in Figure 1b, was dissolved in methanol, filtered through a 0.45-µm membrane, and subjected to HPLC using an Agilent 1100 Series system (Milford, MA, USA). Standard phenolic acids were prepared in methanol (99.8%) at a concentration of 2 mg/mL. A Zorbax SB-C18 column (4.6 × 250 mm, 5 µm) was employed, with a mobile phase consisting of 6% acetic acid in 2 mM sodium acetate and acetonitrile, eluted via a gradient. The acetonitrile concentration increased from 0% to 15% over the first 45 min, then from 15% to 30% over the next 15 min, from 30% to 50% for 5 min, and finally from 50% to 100% for the last 5 min, resulting in a total separation time of 70 min. Ultraviolet detection was performed at 280 nm, with a flow rate of 1 mL/min. The injection volume was 20 µL.

### 4.6. Preparation of Washed Platelets and Plasma from Human Platelet-Rich Plasma (PRP)

Washed human platelets were prepared from platelet-rich plasma (PRP), obtained from healthy volunteers who had not taken medications or dietary supplements known to affect platelet function, including antiplatelet agents and anticoagulants, for at least 7 days prior to blood collection. However, donor demographic information, including the number of donors, sex, and age range, was anonymized and managed exclusively by the blood center and was therefore not available to the investigators.

Donors were screened through routine clinical examinations at the Korean Red Cross Blood Center (KRBC, Changwon, Republic of Korea), and blood collection was performed with informed consent and approval (Safety Supervisor Team-621-2015.02.26) from the institutional ethics committee.

PRP—anticoagulated with an acid–citrate–dextrose solution (0.8% citric acid, 2.2% sodium citrate, and 2.45% glucose)—was centrifuged for 10 min at 125× *g* to remove residual red and white blood cells, followed by a second centrifugation for 10 min at 1300× *g* to isolate platelet pellets and plasma.

The platelet pellets were washed twice with a buffer solution (138 mM NaCl, 2.7 mM KCl, 12 mM NaHCO_3_, 0.36 mM NaH_2_PO_4_, 5.5 mM glucose, and 1 mM disodium ethylenediaminetetraacetate [Na_2_EDTA]; pH 6.5). They were subsequently resuspended in a suspension buffer (138 mM NaCl, 2.7 mM KCl, 12 mM NaHCO_3_, 0.36 mM NaH_2_PO_4_, 0.49 mM MgCl_2_, 5.5 mM glucose, and 0.25% gelatin; pH 6.9) to achieve a final concentration of 5 × 10^8^ platelets/mL. All procedures were conducted at 25 °C to prevent platelet aggregation owing to low temperatures. The washed platelets were used to assess the effects of OJWE on platelet aggregation, Ca^2+^ mobilization, and TXA_2_ production, while the plasma was utilized to evaluate the impact of OJWE on blood coagulation parameters, specifically PT and APTT.

### 4.7. Animals and Administration

We investigated the ex vivo effects of OJWE using male Sprague–Dawley rats (200–250 g; 7 weeks old) supplied by Hyo-Chang Science (Dae-Gu, Kyungpook, Republic of Korea). The rats were categorized into two groups: an OJWE-nontreated control group (n = 5) and an OJWE-treated group (n = 5). Prior to the experiment, the rats were housed individually in stainless steel cages with ad libitum access to water and a standard pellet diet (Sam Yang Oil and Fat Feed Co., Ltd., Seoul, Republic of Korea) for 1 week in an enclosure maintained at 24 °C and 55% ± 5% humidity to acclimate to their environment. Artificial lighting was provided on a 12-h cycle from 7:00 a.m. to 7:00 p.m. OJWE was administered orally at doses of 1 and 5 mg per day for 30 days. The animal experiments were approved by the ethical committee for animal experiments at Inje University.

### 4.8. Preparation of Washed Platelets and Plasma from OJWE-Administered Rats

Prior to platelet and plasma preparation, rats were fasted for 24 h. Anesthesia was induced using ethyl ether, followed by laparotomy to collect 10 mL of blood from the abdominal aorta. The blood was subsequently transferred to a plastic centrifuge tube and centrifuged at 150× *g* for 10 min with 3.8% sodium citrate (9:1, *v*/*v*) as an anticoagulant. The supernatant was collected, and the precipitate was recentrifuged at 150× *g* for 10 min. Thereafter, the supernatants were combined and centrifuged at 100× *g* for 10 min. The final separation of platelets and plasma occurred after centrifugation at 1100× *g* for 10 min.

The separated platelets were washed twice with a washing buffer (138 mM NaCl, 2.7 mM KCl, 12 mM NaHCO_3_, 0.36 mM NaH_2_PO_4_, 5.5 mM glucose, and 1 mM Na_2_EDTA; pH 6.5). To prevent aggregation, the platelets were subsequently washed twice with a suspending buffer containing gelatin, a platelet stabilizer, to eliminate any residual EDTA. The final concentration was adjusted to 5 × 10^8^ platelets mL^−1^ with the suspending buffer, and all procedures were conducted at room temperature to avoid platelet aggregation [56]. Plasma was used to assess the effects of OJWE on blood coagulation parameters, including PT and APTT.

### 4.9. Measurement of In Vitro Human Platelet Aggregation and Ex Vivo Rat Platelet Aggregation

Collagen was selected as a platelet agonist, as it serves as a primary thrombogenic substrate that potently activates platelets via Ca^2+^ mobilization and TXA_2_ production. In this study, washed human platelets were stimulated with collagen to evaluate the effects of OJWE on platelet aggregation, Ca^2+^ mobilization, and TXA_2_ production.

Platelet aggregation was assessed using the following procedure. Washed platelets (10^8^ platelets/mL) from human and rat sources were preincubated in a silicone-treated glass cuvette at 37 °C while stirring at 1000 rpm for 3 min in the presence of 1 or 2 mM CaCl_2_—with or without test substances such as OJWE, verapamil HCl, aspirin, CGA, or CA—and subsequently stimulated for 5 min with collagen (10 μg mL^−1^) using an aggregometer (Chrono-Log Corporation, Havertown, PA, USA) [57]. Human platelet aggregation was assessed using three technical replicates (n = 3), while rat platelet aggregation was determined in five biologically independent rats (n = 5).

The aggregation rate was determined by measuring the increase in light transmission, using the platelet suspension buffer as a reference (0% transmission) to establish the baseline for the aggregometer. The above test substances were dissolved in the platelet suspension buffer (pH 7.4). Since platelets aggregate at low temperatures [58], all platelet aggregation studies were conducted at room temperature.

### 4.10. Measurement of In Vitro Human PT and Ex Vivo Rat PT

Human and rat plasma samples (100 μL each) were preincubated in a two-channel coagulator (Behnk Elektronik GmbH and Co. KG, Norderstedt, Germany) cup (catalog number 95-662; BioMerieux, Marcy l’Étoile, France) for 1 min at 37 °C with gentle stirring. PT was recorded as the time interval from the addition of 100 μL of PT reagent to plasma until the formation of a fibrin clot. Human PT was assessed using three technical replicates (n = 3), whereas rat PT was determined in five biologically independent rats (n = 5).

### 4.11. Measurement of In Vitro Human APTT and Ex Vivo Rat APTT

Similarly, human and rat plasma samples (100 μL each) were preincubated in the same coagulator setup at 37 °C for 1 min with gentle stirring. Thereafter, 100 μL of APTT reagent was added and incubated for an additional 3 min at 37 °C, followed by the immediate addition of 100 μL of 25 mM CaCl_2_. APTT was measured as the time taken to form a fibrin clot. Human APTT assessed three technical replicates (n = 3), while rat APTT was determined in five biologically independent rats (n = 5).

### 4.12. Determination of [Ca^2+^]_i_ In Vitro

PRP was incubated with 5 μM Fura-2 AM, a Ca^2+^ probe, at 37 °C for 60 min. Owing to its light sensitivity, the tube containing PRP and Fura-2 AM was wrapped in aluminum foil during the loading process. The Fura-2 AM-loaded washed platelets were prepared as described earlier, and 10^8^ platelets/mL were preincubated for 3 min at 37 °C with or without OJWE in the presence of 2 mM CaCl_2_. They were subsequently stimulated with collagen (10 μg mL^−1^) for 5 min to assess [Ca^2+^]_i_. The fluorescence intensity of Fura-2 binding to cytosolic Ca^2+^ was measured using a spectrofluorometer (SFM 25; Bio-Teck Instrument, Arcugnano, Italy), with excitation wavelengths alternating every 0.5 s between 340 and 380 nm, and the emission wavelength set at 510 nm. [Ca^2+^]_i_ values were determined using three technical replicates (n = 3) and calculated by the change in fluorescence intensity according to the Grynkiewicz method [59].

### 4.13. Measurement of TXB_2_ In Vitro

Washed platelets (10^8^ platelets mL^−1^) were pre-incubated for 3 min with or without OJWE, aspirin, CA, or CGA in the presence of 2 mM CaCl_2_, and subsequently activated with 10 μg mL^−1^ of collagen. Reactions were terminated after 5 min by adding ice-cold 5 mM EDTA and 0.2 mM indomethacin. TXB_2_, a stable metabolite of TXA_2_, was quantified using a Synergy HT multi-model microplate reader (BioTek Instruments, Winooski, VT, USA) with a TXB_2_ EIA kit. TXB_2_ was determined using three technical replicates (n = 3).

### 4.14. Statistical Analyses

To determine the significance of differences among experimental groups, data are expressed as the mean ± standard deviation (SD). Statistical analyses were conducted using one-way analysis of variance followed by the Newman–Keuls post hoc test when appropriate. All analyses were performed using GraphPad Prism 5 (GraphPad Software Inc., San Diego, CA, USA). Significance levels were set at * *p* < 0.05, ** *p* < 0.01, *** *p* < 0.001, and **** *p* < 0.0001.

## 5. Conclusions

OJWE inhibits collagen-induced platelet activation by suppressing Ca^2+^ mobilization and TXA_2_ production, with enhanced effects observed when combined with verapamil, aspirin, CA, and CGA. Ex vivo dietary intake exerts a modest, dose-related antiplatelet effect without altering coagulation times, indicating selective modulation of platelet pathways without significant impact on hemostasis. These findings underscore *O. javanica* phenolics as food-derived modulators of platelet function with potential cardiovascular implications. Future research should (i) evaluate phenolic metabolites as functional biomarkers, (ii) assess interindividual variability in dietary responsiveness, and (iii) validate translational significance through controlled dietary or clinical intervention studies.

## Figures and Tables

**Table 1:** Effects of OJWE administration on daily feed intake, body weight gain, and feed efficiency ratio in rats.

Group	Feed Intake (g Day^−1^)	Body Weight Gain (g Day^−1^)	FER
Control	24.3 ± 1.5	4.3 ± 0.4	0.176 ± 0.27
OJWE (1 mg day^−1^)	26.2 ± 2.1 *	4.0 ± 0.4	0.153 ± 0.19 *
OJWE (5 mg day^−1^)	26.8 ± 0.7 *	4.4 ± 0.1	0.160 ± 0.14 *

FER, feed efficiency ratio = body weight gain (g day^−1^)/feed intake (g day^−1^). OJWE, hot water extract from *O. javanica*. Data are given as mean ± SD (*n* = 5). * *p* < 0.05 versus control.

## Data Availability

The original contributions presented in this study are included in the article/Supplementary Materials. Further inquiries can be directed to the corresponding authors.

## Supplementary Materials

The following supporting information can be downloaded at https://www.mdpi.com/article/10.3390/ijms27125326/s1.

## Abbreviations

The following abbreviations are used in this manuscript:

AA	arachidonic acid
APTT	activated partial thromboplastin time
CA	caffeic acid
[Ca^2+^]_i_	intracellular calcium concentration
CGA	chlorogenic acid
COX-1	cyclooxygenase-1
CVD	cardiovascular disease
EDTA	ethylenediaminetetraacetic acid
EIA	enzyme immunoassay
FER	feed efficiency ratio
Fura-2 AM	Fura-2 acetoxymethyl ester
GA	gallic acid
GAE	gallic acid equivalent
HPLC	high-performance liquid chromatography
IACUC	Institutional Animal Care and Use Committee
MLCK	myosin light chain kinase
OJWE	Oenanthe javanica hot water extract
PLA_2_	phospholipase A_2_
PLCγ2	phospholipase Cγ2
PRP	platelet-rich plasma
PT	prothrombin time
SD	standard deviation
TXA_2_	thromboxane A_2_
TXB_2_	thromboxane B_2_
