# Supplementary material for: Polyphenol-Rich Oenanthe javanica as a Cardioprotective Functional Food Candidate Exhibiting Antiplatelet Activity via Suppression of Ca2+ Mobilization and Thromboxane A2 Production
Source: Int J Mol Sci. 2026 Jun 12;27(12):5326. doi: 10.3390/ijms27125326 (PMC13299637; doi:10.3390/ijms27125326)
Supplement: Supplementary file 1 [file ijms-27-05326-s001.zip › ijms-4356778-supplementary.pdf]

Table S1 Total phenol content and extraction yield of OJWE

Sample	Extraction solvent	Yield (g)	Yield (%)	Total phenol content (mg GAE g-OJWE^{-1})
O. javanica (1 kg)	Hot water (OJWE)	22.82	2.3	30.1 ± 1.5

GAE, gallic acid equivalent. Data are presented as mean ± S.D. (n = 3).

Table S2 Retention times of phenolic compound standards and OJWE fractions on HPLC

Fraction	Gallic acid (GA)	Caffeic acid (CA)	Chlorogenic acid (CGA)
Standard	3.99 ± 0.14	14.74 ± 0.60	17.75 ± 0.09
Soluble phenolic acid ester (SPAE)	4.14 ± 0.14	−	17.57 ± 0.14
Free phenolics (FP)	4.14 ± 0.14	14.14 ± 0.14	17.57 ± 0.14

Data are presented as retention time (min), mean ± S.D. (n = 3). −, not detected.

Table S3 Analysis and content of phenolic compounds in OJWE

Fraction	Compound	Peak area (h × w/2)	① Standard corresponding amount (µg)	② Amount in SPAE / FP (µg)	③ Injection amount (mg)	④ Content in OJWE (µg mg-OJWE^{-1})
**Standard**	GA	0.375	0.956	−	−	−
	CA	0.270	0.688	−	−	−
	CGA	0.400	1.020	−	−	−
**Soluble phenolic acid ester (SPAE)**	GA	0.160	−	0.408	0.02	20.40
	CGA	0.980	−	2.499	0.02	124.95
**Free phenolics (FP)**	GA	0.128	−	0.325	0.02	16.25
	CA	0.375	−	0.955	0.02	47.75
	CGA	0.595	−	1.517	0.02	75.85
**Total content in OJWE**	**GA**	−	−	−	−	**36.65**
	**CA**	−	−	−	−	**47.75**
	**CGA**	−	−	−	−	**200.80**

① = (Each standard peak area / sum of all standard areas [0.375 + 0.270 + 0.400 = 1.045]) × amount of standard injected (20 µg). ② = Peak area of each compound × ① / standard peak area of that compound. ③ = Amount of SPAE or FP fraction injected onto HPLC. ④ = ② / ③. h, height of peak; w, width of peak at half-height. GA, gallic acid; CA, caffeic acid; CGA, chlorogenic acid. −, not applicable. Total GA = SPAE (20.40) + FP (16.25) = 36.65 µg mg-OJWE $^{-1}$; Total CGA = SPAE (124.95) + FP (75.85) = 200.80 µg mg-OJWE $^{-1}$.

Table S4 Estimated 30-day intake of caffeic acid and chlorogenic acid based on administered OJWE doses in rats

Animal group	Caffeic acid intake (mg, 30 days)	Chlorogenic acid intake (mg, 30 days)
Control (no OJWE)	0	0
OJWE (1 mg day^{-1})	1.425 ①	6.024 ③

OJWE (5 mg day^{-1})	7.125 ②	30.12 ④

① CA 47.75 μg mg-OJWE $^{-1}$ × OJWE 1 mg day $^{-1}$ × 30 days = 1.425 mg. ② CA 47.75 μg mg-OJWE $^{-1}$ × OJWE 5 mg day $^{-1}$ × 30 days = 7.125 mg. ③ CGA 200.8 μg mg-OJWE $^{-1}$ × OJWE 1 mg day $^{-1}$ × 30 days = 6.024 mg. ④ CGA 200.8 μg mg-OJWE $^{-1}$ × OJWE 5 mg day $^{-1}$ × 30 days = 30.12 mg. CA, caffeic acid; CGA, chlorogenic acid; OJWE, Oenanthe javanica hot water extract. Contents of CA and CGA derived from Table S3; CGA represents the sum of soluble phenolic acid ester and free phenolics fractions.
